# The Link Between Autonomic Nervous System and Rheumatoid Arthritis: From Bench to Bedside

**DOI:** 10.3389/fmed.2020.589079

**Published:** 2020-12-07

**Authors:** Francesca Ingegnoli, Massimiliano Buoli, Flavia Antonucci, Lavinia Agra Coletto, Cecilia Maria Esposito, Roberto Caporali

**Affiliations:** ^1^Division of Clinical Rheumatology, Gaetano Pini Hospital, Milan, Italy; ^2^Department of Clinical Sciences and Community Health, Research Center for Adult and Pediatric Rheumatic Diseases, Università degli Studi di Milano, Milan, Italy; ^3^Department of Neurosciences and Mental Health, Fondazione Istituto di Ricovero e Cura a Carattere Scientifico (IRCCS) Ca'Granda Ospedale Maggiore Policlinico, Milan, Italy; ^4^Department of Pathophysiology and Transplantation, Università degli Studi di Milano, Milan, Italy; ^5^Department of Medical Biotechnology and Translational Medicine (BIOMETRA), Università degli Studi di Milano, Milan, Italy

**Keywords:** vagus nerve, central nervous system, autonomic nervous system, mood disorder, rheumatoid arthritis, depression, therapy

## Abstract

Neuronal stimulation is an emerging field of research focused on the management and treatment of various diseases through the reestablishment of physiological homeostasis. Electrical vagus nerve stimulation has recently been proposed as a revolutionary therapeutic option for rheumatoid arthritis (RA) in combination with or even as a replacement for conventional and biological drugs. In the past few years, disruption of the autonomic system has been linked to RA onset and activity. Novel research on the link between the autonomic nervous system and the immune system (immune-autonomics) has paved the way for the development of innovative RA management strategies. Clinical evidence supports this approach. Cardiovascular involvement, in terms of reduced baroreflex sensitivity and heart rate variability-derived indices, and mood disorders, common comorbidities in patients with RA, have been linked to autonomic nervous system dysfunction, which in turn is influenced by increased levels of circulating pro-inflammatory cytokines. This narrative review provides an overview of the autonomic nervous system and RA connection, discussing most of the common cardiac and mental health-related RA comorbidities and their potential relationships to systemic and joint inflammation.

## Introduction

**Figure 1:**
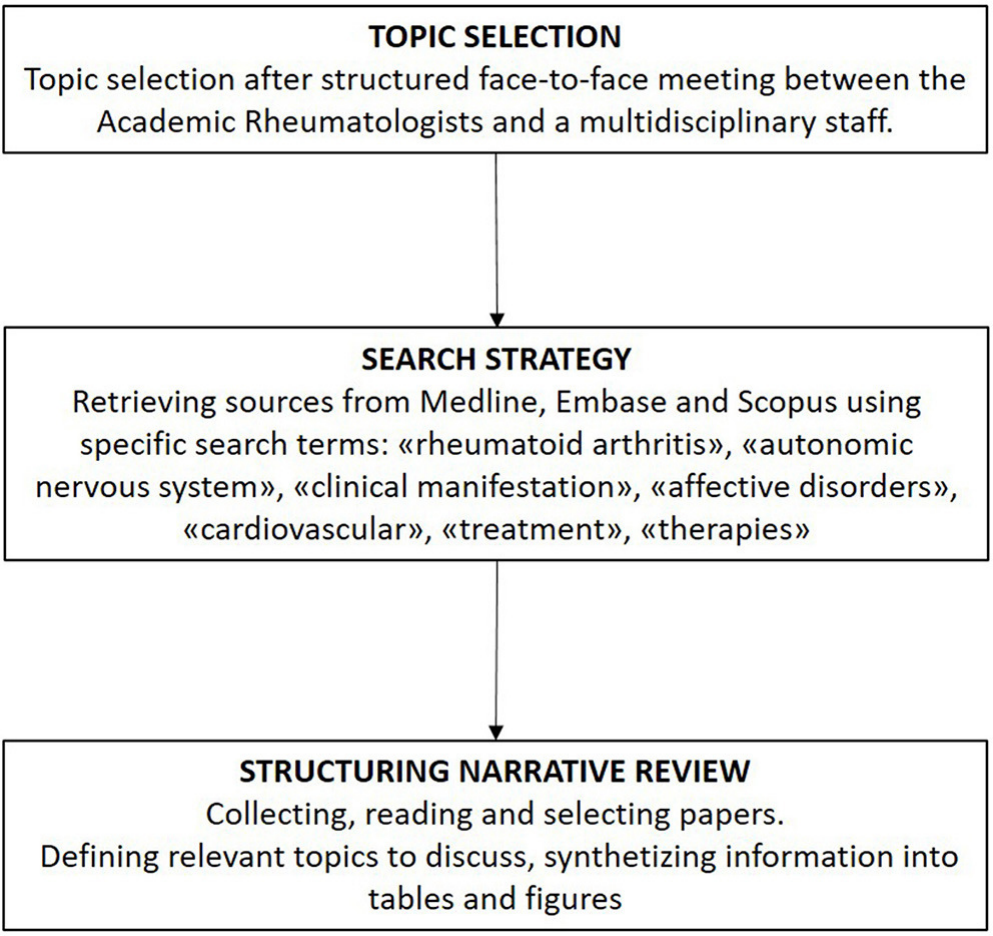
The flow diagram of the narrative review process.

Rheumatoid arthritis (RA) is a chronic autoimmune inflammatory disease leading to progressive joint damage and associated with vascular, metabolic, and psychological comorbidities. RA is considered to pose a major global public health challenge, as its overall prevalence and incidence rates are increasing worldwide (1). Fundamentally, it is of paramount importance to reduce the future burden of this disease through the pursuit of innovative RA treatments that are driven mainly by increasing knowledge of its pathophysiology. An emergent field in this context is the study of autonomic nervous system (ANS) imbalance observed in association with many immune-mediated inflammatory diseases, comprising RA, systemic lupus erythematosus, systemic sclerosis, and inflammatory bowel diseases (2–4). The immune system and ANS can express and respond to numerous common regulatory molecules (e.g., glucocorticoids, cytokines, neuropeptides, and neurotransmitters), which constitute the molecular basis of a complex bidirectional response to homeostasis perturbations induced by infection or inflammation. These findings have led to an innovative research field in RA focused on the emerging concept of “immuno-autonomics,” reflecting the anatomical and functional linkage of the immune system to the nervous system (5). With the understanding of the relevance of this connection, we may consider if and where the ANS is disrupted in RA and hence which therapeutic implications might be relevant. Whether ANS impairment is the result of chronic inflammation or a primitive alteration that affects immune system functioning, disease onset, and severity remains to be established. In this narrative review (Figure 1) (6), we discuss and review the ongoing progress in this field, focusing mainly on current knowledge of the potential connections between ANS and the pathogenesis and clinical manifestations of RA. We then consider the potential innovative therapeutic prospects that target this pathway.

## Relevance of the Autonomic Nervous System to the Pathogenesis of Rheumatoid Arthritis

**Figure 2:**
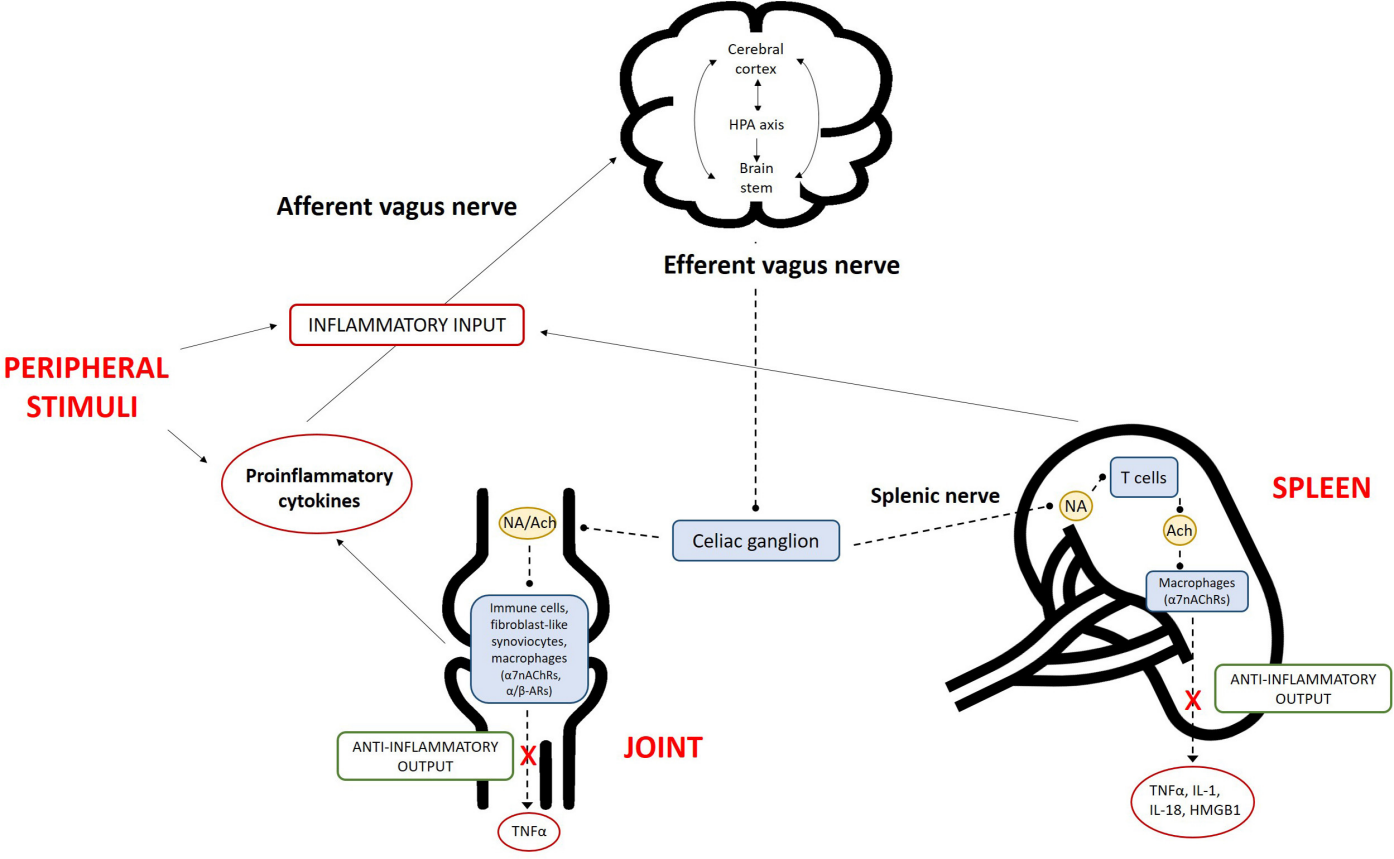
Overview of the inflammatory reflex in rheumatoid arthritis (RA). The central nervous system (CNS) detects peripheral inflammation through the afferent vagus nerve and pro-inflammatory cytokines. The signals act at different levels via (i) activation of the hypothalamic–pituitary–adrenal (HPA) axis, which reduces inflammation; (ii) the cerebral cortex; and (iii) nuclei in the brain stem. The efferent branch of the vagus nerve is activated via the activation of alpha-7 subunit of nicotinic acetylcholine receptors (α7nAChRs) on macrophages, resulting in decreased cytokine production. This mechanism is called the “cholinergic anti-inflammatory pathway.” The combination of inflammation in peripheral tissues, signaling of this information to the brain, and the subsequent efferent neuronal response comprises the “inflammatory reflex.” The activation of α7nAChRs on macrophages can occur locally in the RA joint or in the spleen. The sympathetic fibers of the splenic nerve are activated in the celiac ganglion, leading to noradrenaline (NA) production in the spleen. NA binds to α/β-adrenergic receptors (α/β-ARs) on choline acetyltransferase-positive T cells, which then produce the anti-inflammatory parasympathetic neurotransmitter acetylcholine (Ach), in turn activating α7nAChRs and leading to the reduced production of pro-inflammatory cytokines. In the joint, the activation of a7nAChRs on fibroblast-like synoviocytes or other immune cells also reduces cytokine production. TNF, tumor necrosis factor; IL, interleukin; HMGB1, high mobility group box 1.

The ANS operates through visceral reflex arcs mediated by cholinergic and catecholaminergic signaling (Figure 2). Communication between the ANS and the immune system occurs in two main ways: (i) via direct innervation of the lymphoid organs by the efferent sympathetic nervous system (SNS) [postganglionic noradrenergic fibers innervate the bone marrow, thymus, spleen, and lymph nodes by liberating noradrenaline (NA), neuropeptides (substance P, somatostatin, vasoactive intestinal peptide, and neuropeptide Y), neurokinins, and opioids whose receptors are expressed on immune cells] and (ii) by indirect humoral action mediated by NA (liberated into the bloodstream after medullary activation by the SNS) and steroids [due to activation of the hypothalamic–pituitary–adrenal (HPA) axis neuroendocrine response] (7, 8).

Via beta-2 adrenergic receptors, NA inhibits lymphocyte proliferation, the release of pro-inflammatory cytokines [interleukin (IL)-2, IL-12, interferon gamma, and tumor necrosis factor (TNF)-α], chemotaxis, the activity of natural killer cells and T lymphocytes, and the production of antibodies by B cells. It also stimulates anti-inflammatory cytokines (IL-4, IL-5, and IL-10). Not only postganglionic fibers but also lymphocytes can release NA and acetylcholine, thereby regulating some immune functions in autocrine and paracrine ways (7, 8).

Conversely, the immune system is capable of communicating with the nervous system, mainly via HPA axis activation by pro-inflammatory cytokines and afferent vagal fibers. Neurons express pattern recognition receptors, including toll-like and cytokine receptors (e.g., TNF-R and type 1 IL-1 receptors) (9, 10). This dialog extends beyond the HPA axis; cytokines also directly influence the cerebral cortex and primitive brain (the limbic system, brain stem, and hypothalamus). Cerebral manifestations such as fever, illness, attention deficit, anorexia, and disrupted interaction with external stimuli are examples of their effects during the acute phase of immune activation. In efforts to discern the physiology and pathology of RA, the identification of specific reflexes and pathways is fundamental. A “cholinergic anti-inflammatory pathway,” named after its principal mediator acetylcholine, has been described; its identification has led to the understanding of the “inflammatory reflex” (11). This neural circuit, like other visceral reflex arcs, includes afferent and efferent arms. Vagus afferent fibers are activated peripherally by products of inflammation and then the signal travels to the nucleus tractus solitarius; once the stimulus has been relayed and integrated by other nuclei in the brain stem or hypothalamus (e.g., the rostral ventrolateral medulla and locus coeruleus) (12, 13), the efferent signal leaves the nucleus ambiguus and dorsal motor nucleus and travels back through the vagus nerve until it reaches the celiac ganglia (13, 14). There, preganglionic cholinergic fibers form synapses with adrenergic interneurons, whose splenic fibers end up in the white pulp of the spleen and interact, via NA release, with beta-2 adrenergic receptors of a specific T cell subset. The latter is distinguished by the expression of choline acetyltransferase and when activated induces acetylcholine biosynthesis and release, which in turn stimulates macrophages of the red pulp binding alpha-7 subunit of nicotinic acetylcholine receptors (α7nAChRs) toward an anti-inflammatory immune response (15, 16). The anti-inflammatory signal may bypass the vagus efferent fibers, traveling from the preganglionic fibers of the sympathetic chain directly to the interneurons of the celiac ganglia.

Thus, under basal conditions, the vagus nerve senses the precise location of nascent inflammation and rapidly tonically dampens the overactivation of the innate immune response (17). As the major regulator of the peripheral nervous system (PNS), the vagus modulates inflammation in many anatomical regions, comprising the liver (14), heart (18), pancreas (19), and gastrointestinal tract (20, 21).

The establishment and maintenance of a chronic response involves humoral neuroendocrine mechanisms. This cooperation not only affects innate immunity but also modulates cell trafficking and the lymphoid architecture, ultimately leading to the regulation of humoral immunity and possibly the prevention of T cell-mediated tissue damage (22, 23).

Autonomic imbalance seems to be an early finding, rather than the result of chronic inflammation, in patients with RA. In a prospective cohort study, in which ANS activity was assessed using a validated method via the measurement of subjects' resting heart rate (HR) and heart rate variability (HRV), individuals at risk of RA who subsequently developed arthritis had significantly higher resting HRs than healthy subjects (24). This finding is in agreement with those from other studies, which reflect reduced PNS activity and hence an impaired inflammatory reflex, in patients with RA (25, 26). The early impairment of the PNS in RA, even before the fulfillment of the disease classification criteria (27), is consistent with the correlation between increased inflammatory status and decreased parasympathetic activity observed in healthy subjects in large observational studies (28–30). In detail, levels of inflammatory markers [C-reactive protein (CRP) and IL-6] are inversely related to HRV in young adults (29), and circulating TNF level is an independent predictor of depressed HRV (31), reinforcing the concept of a complex dialog between immunity and the ANS.

As the PNS and SNS work together, we would expect the SNS to be less active when the PNS is impaired. Contrary to this expectation, the SNS has been found to be overactive in patients with RA and high NA levels (25), rendering comprehension of its pathogenetic role difficult (32). One explanation could involve a difference in intracellular signaling (5). Splenic beta-2 adrenergic receptors usually promote a T helper (Th) 2 and T regulatory (Treg) response via G-coupled proteins and protein kinase A pathways. However, the scenario in which the SNS seems to be chronically activated may entail the downregulation of beta-2 adrenergic receptors and a shift toward Th1 and/or Th17 immune responses via mitogen-activated protein kinase pathways (5).

Collectively taken, these data support the critical role of ANS in RA pathogenic network.

## Autonomic Nervous System and Affective Disorders in Rheumatoid Arthritis

Peripheral cytokines profoundly influence neuronal function and brain circuitry. As briefly mentioned above, they reach the brain by different routes and, once there, affect brain function through several mechanisms. They may directly stimulate (i) the central nervous system (CNS) cell population (microglia, astrocytes, and neurons), producing additional cytokines (33); (ii) the HPA axis, resulting in the production of corticotropin-releasing factor and adrenocorticotropic hormone; and (iii) cortisol, influencing many other physiological processes in the CNS. Cytokines alter the metabolism of several neurotransmitters, including serotonin (34, 35), dopamine (35), and glutamate (36, 37), leading to the decreased production of norepinephrine and the trophic or growth factors that are essential for neurogenesis and neuroplasticity (38–40). Changes in all of these factors and amines may lead to the development of psychiatric disorders, further corroborating the link between cytokine increments and mental health. In many studies, the continuous elevation of IL levels has been correlated with impairments in structures profoundly affected in mood disorders, such as the hippocampal region (41, 42) and other areas of the brain (43–45), as well as changes in functional connectivity (46, 47).

In the CIA model, researchers observed exacerbated symptomatology in association with greater cytokine production in mice lacking α7nAChR, suggesting that nicotinic receptor expression is relevant in RA and that the activation of these receptors would have beneficial effects. Interestingly, cholinergic agonists suppressed inflammatory cytokine production in RA whole blood cultures (48). Choline acetyltransferase expression was observed in fibroblast-like synoviocytes and mononuclear cells in RA and osteoarthritis synovial biopsy samples, suggesting that local acetylcholine production contributes to the regulation of joint inflammation by the aforementioned “cholinergic anti-inflammatory pathway” (49). Although the activation of α7nAChR leads to control of the degree of inflammation, the effects of this receptor's action on central neurons, brain functionality, and related cognitive behaviors have not been examined. In general, results indicate that (i) muscarinic agonist administration, (ii) electrical vagus nerve stimulation (VNS) to activate preganglionic parasympathetic nerves, and (iii) treatment with nAChR agonists can all act systemically (although not necessarily identically) to reduce the production of inflammatory cytokines (presumably mostly by macrophages).

Abundant data support associations between autoimmune diseases and psychiatric disorders, with immune activation identified as the common core feature (50, 51). The prominence of psychiatric comorbidities, including major depressive disorder (MDD), bipolar disorder, and anxiety disorders (ADs), in the presence of autoimmune diseases such as RA supports the theory that affective disorders can be considered to be inflammatory conditions (52).

From an epidemiological point of view, concomitant RA and depression has been reported in 6.8–66.2% of patients (53–62). Risk factors for MDD onset in patients with RA include female sex (63, 64), unmarried status, the lack of sufficient social support (65), a high rate of disability (66), and chronic pain (67). Moreover, the presence of depressive symptoms increases the risk of suicide in subjects, and especially women, with RA (68).

In most described cases, depression appears secondary to RA development (69–72). Several pathogenetic mechanisms have been proposed to explain this association. From a neurobiological perspective, reduced expression of BDNF, but not serum levels of pro-inflammatory cytokines (59) or TNF-α (57), has been found in depressed patients with RA with respect to subjects without depression. In contrast, other studies have documented a relationship between MDD and the inflammatory state, defined by plasma CRP levels (73) or indirectly via clinical indices of RA activity (74). The implication of over-inflammation in the onset of mood symptoms in RA is also supported by the evidence of alterations in the micro-structure of brain white matter in subjects with RA and comorbid depression as a result of vasculitis, ischemic brain lesions, and dots of demyelination (75). In contrast, one study failed to identify shared genetic vulnerability to RA and MDD (76). From a psychological perspective, the RA self-schema construct seems to predict the onset of depression. Interpersonal conflicts seem to increase the risk of depression in patients with RA. Vulnerability to depressive symptoms in patients with RA appears to be related to functional disabilities (59, 77–79) and pain (78, 80–82) caused by the disease, although some researchers failed to find an association between RA activity or severity and depression (56, 81, 83).

Comorbid depression increases social impairment (84, 85) and disability (86) in patients with RA. Functional capacity seems to be more compromised in women than in men with concomitant RA and MDD (63). The occurrence of depression in patients with RA worsens the subjective sensation of pain (87–90), thereby encouraging the abuse of analgesic compounds (91, 92). After all, the association between depression and pain sensitivity is already known within the framework of fibromyalgia (93). Of note, the presence of depressive symptoms in musculoskeletal pathologies even reach 81.8% as reported in a recent study (94), where a correlation between the severity of depressive symptoms and fibromyalgia was also identified. Although most studies have focused on the effects of RA on mental health, some authors have hypothesized conversely that depression directly influences RA activity and the number of compromised joints (95–97). In addition, comorbid depression in patients with RA favors the onset of medical complications such as atherosclerosis (98) and myocardial infarction (99), resulting in increased lethality (100, 101). Depressive symptoms also may prevent patients' treatment adherence, thereby worsening the prognosis of RA (96, 102). Taken as a whole, these data indicate that depression worsens the quality of life (90, 103) and general health status (104) of subjects with RA.

Depression is often underestimated in patients with RA, delaying its proper management (94, 105). The treatment of depressive symptoms seems to ameliorate the clinical symptoms of RA (106), although some data contradict the existence of this effect (107). Antidepressants demonstrated to be effective in cases of RA-MDD comorbidity include dothiepin (108) and sertraline (109), eventually combined with cognitive behavioral treatment (107). Non-pharmacological strategies, including yoga, mindfulness meditation, and emotion regulation therapy, have been shown to significantly ameliorate mood symptoms and inflammatory status in patients with RA (110, 111). Finally, immunomodulating drugs, such as anti-TNF-α drugs (105, 112), anti-IL-6 monoclonal antibodies (113), and other biologics (114), appear to ameliorate anxiety and depressive symptoms in patients with RA. Of note, in line with the autonomic dysfunctions already mentioned for RA, VNS was proposed as a treatment for depression in the light of its anti-inflammatory effect (115). This convergence of the efficacy of VNS both for RA and depression opens up extremely promising therapeutic prospective for the treatment of subjects suffering from RA and mood symptoms (116).

ADs have the common psychopathological nucleus of anxiety and partly shared neurobiological abnormalities. They include panic disorder, social phobia (SP) (also known as social anxiety disorder), and generalized anxiety disorder (GAD). Their prevalence in RA ranges from 13 to 70% (53, 55, 65, 78, 117–119) with higher incidence and prevalence compared to the general population (120). Anxious states often occur concomitantly with depressive symptoms (54, 65). SP seems to be more common in individuals with RA than in those with other autoimmune conditions (121, 122). ADs are considered to be a risk factor for the future development of RA (120) and appear to negatively affect its course, worsening functional disabilities (78, 90, 123) and quality of life (90, 103, 124, 125) and changing pain perceptions (82, 89, 126). Moreover, psychological stress and anxiety symptoms were reported to be the most frequent causes of joint symptom exacerbation (127). In support of these clinical data, patients with RA and anxiety were found to have higher serum levels of IL-17 than those without AD (128).

Some authors have hypothesized that ADs in patients with RA are triggered by psychosocial factors, such as pronounced neuroticism, a lower educational level, and poor social support (69). According to this explanation, RA-associated disability in case of AD comorbidity is attributable more to psychological factors implicated in all chronic diseases than to the worsening of symptoms as a result of an increased pro-inflammatory state (129).

Proper AD treatment in patients with RA is essential to prevent functional decline (130) and poor adherence to treatment (131). Some drugs prescribed for RA, such as anti-TNF-α medications, seem to have beneficial effects on anxiety symptoms (112), and their discontinuation is related to the worsening of anxiety (132).

## Autonomic Nervous System and Cardiovascular Manifestations in Rheumatoid Arthritis

Cardiovascular autonomic dysfunction in RA has been investigated by measuring heart rate (HR) and heart rate variability (HRV) (24). The link between the cardiovascular system and ANS imbalance in RA could partly explain the well-documented augmented cardiovascular disease and RA-related mortality, not fully justified by traditional risk factors.

It is well known how chronic inflammation influences the development of cardiovascular disease (CVD) by promoting atherosclerosis, myocardial remodeling, and insulin resistance and by modifying lipid levels and function and oxidative stress (133). Furthermore, multimodality imaging is useful to identify high-risk patients who benefit from preventive strategies or treatment intervention (134); the link between brain and heart damage in RA has been documented by magnetic resonance imaging (135).

Fundamentally, we have limited understanding of the mechanisms underpinning the connections between ANS and CVD. An extensive assessment of ANS by means of HR, cardiac/sympathetic baroreflex, and muscle sympathetic nerve activity (MSNA) was carried out in 30 RA patients (normo- and hypertensive) matched with a control group. Regardless the presence of hypertension, HR and sympathetic activity were increased and cardiac baroreflex sensitivity was reduced in RA patients, while sympathetic baroreflex sensitivity was preserved. Moreover, these findings were correlated with pain (VAS) and inflammation (CPR). Hence, a heightened sympathetic outflow was confirmed along with a reduced arterial baroreflex control of the heart both linked directly to RA symptoms, excluding the bias hypertension, a common cardiovascular risk factor (136).

Although data suggest that ANS imbalance is detrimental for cardiovascular disease, including cardiac arrhythmias, hypertension (137), and increased mortality (138, 139), breaking through the original trigger of this vicious circle with ultimate damage of the cardiovascular system is particularly difficult. Moreover, patients with RA without clinical cardiovascular disease have reduced left ventricular systolic function assessed by global longitudinal strain by speckle-tracking echocardiography, and it is related with disease activity (140).

Another study showed how a reduced coronary microvascular perfusion in RA patients, in terms of subendocardial viability ratio, associates with markers of disease activity and classical CVD risk factors, including heart rate. This reinforces the hypothesis that inflammation, by the stimulation of ANS, may impair myocardial perfusion (141).

Finally, according to previous literature, the evidence of cardiovascular autonomic dysfunction might be used to identify patients with RA who respond better to TNF inhibitors (25, 142). In particular, a low parasympathetic outflow and a high sympathetic outflow were associated with a poor anti-TNF response, making HRV a promising useful predictor of outcome, with great benefits for overall costs and quality-adjusted life-years (QALYs) (143).

Furthermore, cardiovascular autonomic imbalance associates with comorbidities including depressive and anxiety disturbances compared to healthy controls (144). Finally, looking at a therapeutic prospective, non-pharmacological intervention such as exercise training, given its well-known benefits on cardiovascular autonomic function, might help restore ANS imbalance, increasing vagal-related indices in RA with consequent potential benefit on pain and inflammation (145). Likewise, ANS imbalance could be partially restored by optimizing pain management (146).

## Implications for Treatment and Future Perspectives

Altered ANS parameters have been associated with increased Disease Activity Score-28 (DAS-28), CRP, and erythrocyte sedimentation rate (25, 147). Seropositive RA patients are more prone to ANS dysfunction and more likely to experience the amelioration of cardiovascular autonomic neuropathy when treated with synthetic and biologic disease-modifying antirheumatic drugs (DMARDS) than seronegative patients with RA (148, 149). Thus, seropositivity, along with disease activity and pro-inflammatory cytokine levels, is predictive of autonomic dysfunction (150). Moreover, in patients with RA and ankylosing spondylitis, HRV was shown to predict the clinical response to anti-TNF therapy; specifically, patients with more vagal activity responded better to this treatment (142). Biologic, and to a lesser extent synthetic, DMARDs significantly improved autonomic neuropathy, including all of its parasympathetic, sympathetic, and sudomotor components, in patients with RA and ankylosing spondylitis (148).

Multiple strategies can be used to achieve these goals. VNS can dampen inflammation early in a discrete and localized manner. Administration of the specific agonist of splenic α7nAChR acts on downstream of the pathway. Antagonization of the SNS is a possible alternative that should be explored. As shown in Table 1, four clinical trials examining VNS have been published (151–154), and one of these has been presented at the last EULAR e-congress (153). As they involved the use of different devices, modalities, timing, and stimulation sites, comparison of their results is (152) not feasible, but the researchers observed significant and rapid declines in the production of pro-inflammatory cytokines such as TNF-α, IL-6, and IL-1b after VNS in patients with epilepsy, those with RA, and healthy subjects (151, 152). In the first trial, VNS was performed directly via an electronic device (Cyberonics) implanted under general anesthesia. Three helical coiled cuffs around the vagus nerve and a lead were then tunneled subcutaneously from the neck and connected to a pulse generator placed in a subcutaneous pocket on the chest wall (151). The only adverse events reported were mild/moderate and related to the surgical implanting approach. In the most recent trial, a MicroRegulator device was implanted on the left cervical vagus nerve. The device was well tolerated without adverse events. The sample size was small, but VNS reduced disease activity in 50% of the highly drug refractory RA patients (154). In the other two trials, a non-invasive device consisting of a hand-held probe with a tip producing radial displacement in a circular pattern at the probe was used. Transcutaneous auricular VNS consisted of the application of electrical signals to the cutaneous territory supplied by the auricular branch of the vagus nerve at the cymba concha (152, 153). In all trials, disease activity was rapidly attenuated after VNS in patients with active RA (biologic-naïve subjects and those for whom multiple treatments had failed).

**Table 1:** Targeting vagus nerve by electrical stimulation in rheumatoid arthritis.

**Clinical trial**	**Study population**	**Device and regimen**	**Primary endpoint**	**Safety data**	**Length**
Koopman 2016 (151)	17 active RA (7 naïve to bDMARDs and 10 bDMARDs failure) Open label	Cyberonics device around the cervical vagus nerve; implantation under general anesthesia.Day 0–28 daily stimulation with escalating intensity	DAS28-CRP at week 6 (baseline 6.05 ± 0.18 at day −21 vs. 4.16 ± 0.39 at day 42, *p* < 0.001)	No serious adverse events, mild/moderate events associated with implanting device	12 weeks
Addorisio 2019 (152)	9 active RA Randomized cross-over	Non-invasive vibrotactile auricular stimulationStimulation twice daily for 2 days	DAS28-CRP at week 1 (baseline 4.19 ± 0.33 vs. 2.79 ± 0.21 at day 7, *p* < 0.01)	No adverse events	1 week
Marsal 2020^*^ (153)	30 active RA Open label	Non-invasive vibrotactile auricular stimulationData on regimen stimulation not available	DAS28-CRP at week 12–1.40 (*p* < 0.01)	1 device superficial skin abrasion resolved without intervention	12 weeks
Genovese 2020 (154)	14 active RA with insufficient response ≧ 2 b/tsDMARDs Stage 1 open label; stage 2 randomized, multicentre, sham-controlled trial	Miniaturized VNS device implanted on the left cervical vagus nerve	Safety and tolerability of the implantation surgical procedure, device and the active treatment	Horner's syndrome, procedural pain, contact dermatitis, vocal cord paralysis. All adverse events were related to the surgical procedure and resolved without permanent clinically significant sequelae	12 weeks

*RA, rheumatoid arthritis; DAS, disease activity score; CRP, C-reactive protein; bDMARD, biological disease-modifying anti-rheumatic drug; tsDMARDS, targeted synthetic DMARDs; VNS, vagus nerve stimulation*.

* *Results only available from the congress abstract*.

Considering the consistent percentage of patients who do not respond at all to the available drugs or have unsatisfactory responses, have gradual loss of responsiveness over time, or have drug-related adverse events, a non-pharmacological approach such as bioelectronics might be a useful further tool in the successful expansion of the therapeutic armamentarium of RA.

Bioelectronic medicine, based on neuromodulation of the nervous system restoring organ and immune system function, is a new potential tantalizing and promising field. In particular, the use of non-invasive devices has lesser adverse effects than drugs and has greater treatment adherence. Naturally, the therapeutic potential of immuno-autonomics has been further demonstrated, and the optimal neurostimulation parameters to achieve and maintain significant clinical changes are still unknown.

## Conclusion

The innovative view of the ANS and RA axis described here allows for the development of new strategies for RA management. The ANS influences key aspects of joint pathophysiology and may be a target of novel therapeutic approaches. The emergence of immuno-autonomics and encouraging results of clinical trials could have extraordinary implications for RA monitoring and treatment. Further research in this field will be useful to understand the full potential of therapeutic models based on the brain–joint axis, with the integration of different findings.

## Author Contributions

All authors listed have made a substantial, direct and intellectual contribution to the work, and approved it for publication.

## Conflict of Interest

The authors declare that the research was conducted in the absence of any commercial or financial relationships that could be construed as a potential conflict of interest.
